# Deep Learning-Based Detection and Classification System for Salivary Gland Tumors Using Ultrasound Imaging

**DOI:** 10.3390/biomedicines14081786

**Published:** 2026-08-07

**Authors:** Wei-Chen Hung, Chung-Sheng Wu, Chih-Ming Chang, Wu-Chia Lo, Li-Jen Liao, Ping-Chia Cheng

**Affiliations:** 1 Department of Otolaryngology, Head and Neck Surgery, Far Eastern Memorial Hospital, New Taipei City 22060, Taiwan; davidhon1990515@gmail.com (W.-C.H.); gwuchonson@gmail.com (C.-S.W.); b88401077@ntu.edu.tw (C.-M.C.); lowuchia@gmail.com (W.-C.L.); deniro@mail2000.com.tw (L.-J.L.); 2 Department of Communication Engineering, Asia Eastern University of Science and Technology, New Taipei City 22060, Taiwan; 3 Head and Neck Cancer Surveillance and Research Study Group, Far Eastern Memorial Hospital, New Taipei City 22060, Taiwan; 4 Department of Biomedical Engineering, National Yang Ming Chiao Tung University, Taipei 11221, Taiwan; 5 Graduate Institute of Medicine, Yuan Ze University, Taoyuan 32003, Taiwan; 6 Department of Electrical Engineering, Yuan Ze University, Taoyuan 32003, Taiwan

**Keywords:** salivary gland tumor, ultrasound, deep learning, object detection, object classification

## Abstract

**Objectives**: Ultrasound is the primary modality for salivary gland tumor (SGT) evaluation, yet its reliance on subjective interpretation can lead to diagnostic variance. This study aims to develop and validate a two-stage deep learning system to automate SGT detection and classification. **Methods**: The study compiled a dataset of ultrasound images from patients with pathologically confirmed SGTs across three sequential cohorts: a training set (687 images, 2007–2020), a validation set (78 images, 2021), and a testing set (101 images, 2022). A YOLOv8 model was trained for tumor detection, and a modified ResNet50V2 model was utilized for benign versus malignant classification. The resulting two-stage pipeline was deployed on a local desktop system and further evaluated using two independent datasets: an internal validation set (56 images, 2023) and an external online dataset (57 images). **Results**: On the testing set, the YOLOv8 model achieved a bounding-box precision of 0.94 and a recall of 0.95 for tumor detection. When integrated with the classification model, the two-stage desktop system yielded an accuracy of 84%, sensitivity of 74%, and specificity of 87%. This system maintained comparable performance, demonstrating accuracies of 82% and 81%, sensitivities of 100% and 71%, and specificities of 81% and 86% on the internal and external validation sets, respectively. **Conclusions**: This study introduced a two-stage deep learning desktop system for automated SGT diagnosis. The edge-deployed system may serve as an objective adjunct to conventional ultrasound interpretation, potentially assisting clinicians during preoperative evaluation.

## 1. Introduction

Salivary gland tumors (SGTs) comprise a complex, heterogeneous group of head and neck neoplasms, presenting an annual incidence ranging from 2.5 to 8.6 cases per 100,000 individuals [1,2]. While malignant SGTs constitute less than 20% of these cases, they require more intensive treatment than benign SGTs, underscoring the need for comprehensive treatment planning. Current preoperative assessments include diagnostic imaging and cytopathological examinations. High-resolution ultrasound serves as the primary modality for evaluating SGTs due to its superficial tissue clarity, rapid execution, lack of ionizing radiation, and capability to guide real-time fine-needle aspiration cytology (FNAC). However, standard ultrasound interpretation remains highly operator-dependent and subjective. In our previous work, we developed a subjective ultrasound scoring model for evaluating SGTs [3]. We further compared this score with ultrasound elastography and FNAC. The results indicated that in differentiating between malignant and benign SGTs, the subjective ultrasound score had a sensitivity of 58%, specificity of 89%, and accuracy of 85%, while ultrasound elastography had a sensitivity of 69%, specificity of 70%, and accuracy of 70%, and FNAC had a sensitivity of 74%, specificity of 93%, and accuracy of 91% [4]. Despite the high accuracy, all methods suffered from low sensitivity, even with cytology. This vulnerability, paired with pronounced inter-observer variability among clinical specialists, highlights a critical diagnostic gap. Consequently, establishing an automated, objective, and reproducible diagnostic framework is essential.

Deep learning (DL) techniques are increasingly being applied to medical image analysis, including tasks such as classification, detection, and segmentation [5,6]. In our previous study, we developed a transfer-learning framework utilizing a modified ResNet50V2 architecture that successfully classified SGTs from ultrasound imaging [7]. However, it relied on cropped ultrasound images restricted to the tumor region alone, necessitating an additional step for manual cropping and potentially introducing bias during clinical application. To address this limitation, we explored object detection models for automated tumor region cropping [8,9]. Object detection architectures are broadly categorized into two types: two-stage models, which utilize separate region proposal networks prior to classification, and one-stage models, which map image pixels directly to bounding-box coordinates and class probabilities. While two-stage models historically offer marginal accuracy advantages, their computational complexity introduces significant latency that limits real-time application. Conversely, one-stage models provide rapid inference speeds suitable for edge deployment. Our approach combines an object detection model with our existing classification model, allowing us to achieve both automated tumor detection and subsequent classification.

In this study, we selected YOLOv8, the latest version of the YOLO (You Only Look Once) family, due to its optimized architectural backbone, lightweight size, and rapid inference capability [10,11]. After training YOLOv8, we coupled it with our previously modified ResNet50V2, which resulted in a two-stage detection-then-classification system. Furthermore, to facilitate real-time, edge-side analysis without relying on high-end external graphics processing units (GPUs), we compressed the integrated models using TensorFlow Lite (TFLite) post-training quantization. This optimization converts floating-point weights into efficient 8-bit integers, minimizing computational overhead, reducing model sizes, and permitting fully offline deployment on a local desktop. This architecture ensures complete data containment, preserving patient data privacy.

This study aimed to develop and validate a two-stage deep learning system to seamlessly integrate object detection and classification to automate SGT analysis in ultrasound images. Its performance was evaluated using both internal and external validation datasets to assess its generalizability across different clinical settings.

## 2. Materials and Methods

### 2.1. Study Design and Patient Selection

The study was conducted at a tertiary medical center, in compliance with the Declaration of Helsinki, and received approval from the institutional ethical review board (IRB No. 112136-E and 115087-E). We reviewed the records of patients who visited our outpatient department between January 2007 and December 2022 and underwent ultrasound examinations for SGT. The ultrasounds were performed by two experienced otolaryngologists using a Toshiba Aplio 500 (Canon Medical Systems, Tochigi-ken, Japan) equipped with a 5–14 MHz linear-array transducer. To be included in the final analysis, patients were required to have complete B-mode ultrasound profiles and a definitive pathological diagnosis obtained via surgical excision or core needle biopsy (CNB), the latter reserved for cases where open surgery was contraindicated. Pathological reports served as the definitive ground truth for classifying tumors into benign or malignant categories. Exclusion criteria eliminated cases with inadequate image quality or uncertain pathological findings.

### 2.2. Data Preparation and Image Annotation

Ultrasound images were retrieved from the picture archiving and communication system (PACS), covering various views of SGTs. We preserved ultrasound images while ensuring all identifiable information was removed to protect patient confidentiality. This process involved the elimination of names, medical record numbers, birth dates, and dates of ultrasound execution. All patient-identifiable information, such as names and medical record numbers, was institutionally anonymized and digitally masked by cropping the region of interest (ROI) containing the B-mode ultrasound frames prior to system input. For effective model establishment and evaluation, the dataset was partitioned into training, validation, and test sets based on the diagnosis period. To mitigate class imbalance, additional ultrasound images were collected from patients with malignant tumors, thereby increasing the representation of malignant cases across all datasets. The training set included 264 unique patients (222 benign and 42 malignant) diagnosed between January 2007 and December 2020, yielding 687 images (475 benign and 212 malignant). The validation set comprised 32 unique patients (26 benign and 6 malignant) diagnosed between January 2021 and December 2021, providing 78 images (54 benign and 24 malignant). The testing set consisted of 43 unique patients (38 benign and 5 malignant) diagnosed between January 2022 and December 2022, contributing 101 images (82 benign and 19 malignant). No patient was included in more than one cohort. Although this patient cohort (2007–2022) overlaps with that of our prior study [7], which evaluated a single-stage classification model on manually cropped images, the present study is methodologically distinct. Here, we present an independent, automated two-stage detection-and-classification framework with a distinct deployment pipeline and validation strategy.

For the object detection training phase, ground-truth bounding boxes were annotated by the same two experienced otolaryngologists. Using labelImg (version 1.8.6), an open-source, Python (version 3.8)-based graphical annotation utility, rectangular boundaries were drawn tightly around the visible perimeter of each tumor. For images exhibiting multiple lesions, all visible tumors were annotated. However, during downstream model deployment, only the bounding box with the highest YOLOv8 confidence score was selected for real-time analysis. Bounding boxes were labeled as ‘tumor’. To facilitate integration with the second-stage classification model, we further assigned class labels according to its pathological diagnoses: benignity as class 0 and malignancy as class 1. To maximize inter-observer reliability, all final bounding boxes were cross-verified and approved by a third otolaryngologist.

### 2.3. Model Establishment

We developed our model using the Python (version 3.10) programming environment on Google Colaboratory (Colab), equipped with an NVIDIA T4 GPU (NVIDIA Corp., Santa Clara, CA, USA). Colab provides complimentary GPU resources and functions as a web-based Jupyter Notebook. YOLOv8 was used for object detection training [12]. Input images were resized to 128 × 128 pixels to minimize spatial redundancy and optimize training stability for edge-side deployment. The YOLOv8 architecture partitions each input image into a grid, with each grid cell responsible for detecting objects whose centers fall within its boundaries. Fully connected layers connect the convolutional backbone to YOLO’s output layer, enabling object localization and classification. For bounding-box prediction, YOLOv8 adopts Distribution Focal Loss (DFL) and Complete Intersection over Union (CIoU) loss. DFL, an enhanced variant of Focal Loss, mitigates class imbalance by adjusting sample weights according to class confidence distribution. CIoU loss, an advanced form of Intersection over Union (IoU) loss, evaluates the accuracy of predicted bounding boxes by considering overlap, center distance, and aspect ratio consistency with ground truth. In this study, the YOLOv8n model was selected for application. YOLOv8n was initialized with COCO-pretrained weights and trained for 300 epochs using the AdamW optimizer (learning rate = 0.002, momentum = 0.9, weight decay = 0.0005) with a batch size of 16 and a 3-epoch linear warm-up. Standard YOLOv8 data augmentations were applied, including HSV color jitter, ±10% translation, ±50% scale, 50% horizontal flip, and mosaic augmentation. The NMS IoU threshold was set to 0.7, and a fixed random seed (seed = 0) was applied to ensure deterministic training. Throughout the 300 epochs, performance was continuously evaluated on the validation cohort to monitor metrics and prevent overfitting. To ensure rigorous evaluation and prevent data leakage, the classification model (modified ResNet50V2) shared the same training (2007–2020) dataset as the YOLOv8 model. After training the detection model, the cropped tumor regions were extracted from the original-sized ultrasound images based on the predicted bounding boxes. For detection post-processing, the confidence threshold was set to 0.1 to maximize candidate recall, while the NMS IoU threshold was kept at 0.7 to suppress overlapping bounding boxes. When multiple candidate boxes were detected, the box with the highest YOLOv8 confidence score was selected for downstream cropping and classification, and cases with no detection were reported as “no tumor detected” rather than being forced into a classification. These cropped regions then underwent histogram equalization to reduce grayscale intensity variations resulting from differences in operator-dependent gain settings. Finally, the processed images were resized to 150 × 150 pixels and forwarded to the modified ResNet50V2 architecture for classification. The previously trained ResNet50V2 classifier was too large for real-time deployment on our edge device. To enable efficient deployment on edge devices, the classification model was further optimized using TFLite quantization. This compression pipeline converted floating-point parameters into 8-bit integers, substantially reducing computational load and inference latency while preserving diagnostic performance. TFLite quantization reduced the classification model from 500 MB to 40 MB (a 92% size reduction), and a paired comparison confirmed a negligible difference in classification accuracy between the full-precision and quantized models. Overall, a two-stage pipeline was developed, consisting of tumor detection followed by benign–malignant classification.

### 2.4. Application Development

To facilitate clinical translation, we developed two distinct interfaces for model deployment, including a web application and a local desktop system. The web application interface was designed for accessibility and ease of use. The interface features a vertical layout with clearly defined sections: a title header (A), an upload function for image submission (B), display of the uploaded filename (C), classification results (D), the original ultrasound image (E), and visualization of the detected tumor region with bounding-box overlay (F). This web-based platform allows clinicians to access the diagnostic tool from any internet-enabled device without requiring local installation. The application code is hosted in a GitHub repository and deployed via the Streamlit platform. However, uploading patient images to the cloud may raise privacy concerns. To address institutional data privacy requirements and support offline, real-time clinical use, a local desktop system was developed using Python Tkinter (version 8.6). This interface features a dual-panel design. The left panel serves as the control center, allowing users to integrate external streaming video from ultrasound machines, perform screen captures from external viewers, or upload static images and videos for analysis. For resource-constrained edge devices, toggle switches allow users to switch from continuous real-time stream processing to single-frame snapshot analysis. The right panel serves as the display area, presenting video streams or uploaded images alongside corresponding analysis results, including the detected tumor boundary and classification output. The final diagnostic inference is performed using the desktop-based application system.

### 2.5. Internal and External Validation

To rigorously evaluate the generalizability of the desktop application, two independent validation datasets were collected. The internal and external validation cohorts each included one representative image per patient. The internal validation set included 52 benign and 4 malignant ultrasound images. Each image was sourced from a patient diagnosed between January 2023 and December 2023 in our hospital. The external validation set comprised 36 benign and 21 malignant ultrasound images. Each image was sourced from a patient whose case was reported on an online case report website (https://www.ultrasoundcases.info, accessed on 22 March 2024). Formal permission was secured to utilize these external cases for research validation.

### 2.6. Statistical Analysis

The diagnostic performance of the integrated desktop system was validated using the testing set and both validation cohorts. Performance metrics calculated include accuracy, sensitivity, specificity, positive predictive value (PPV), and negative predictive value (NPV). Confusion matrices were generated for the validation datasets to evaluate the classification distribution across benign and malignant categories.

## 3. Results

**Figure 1:**
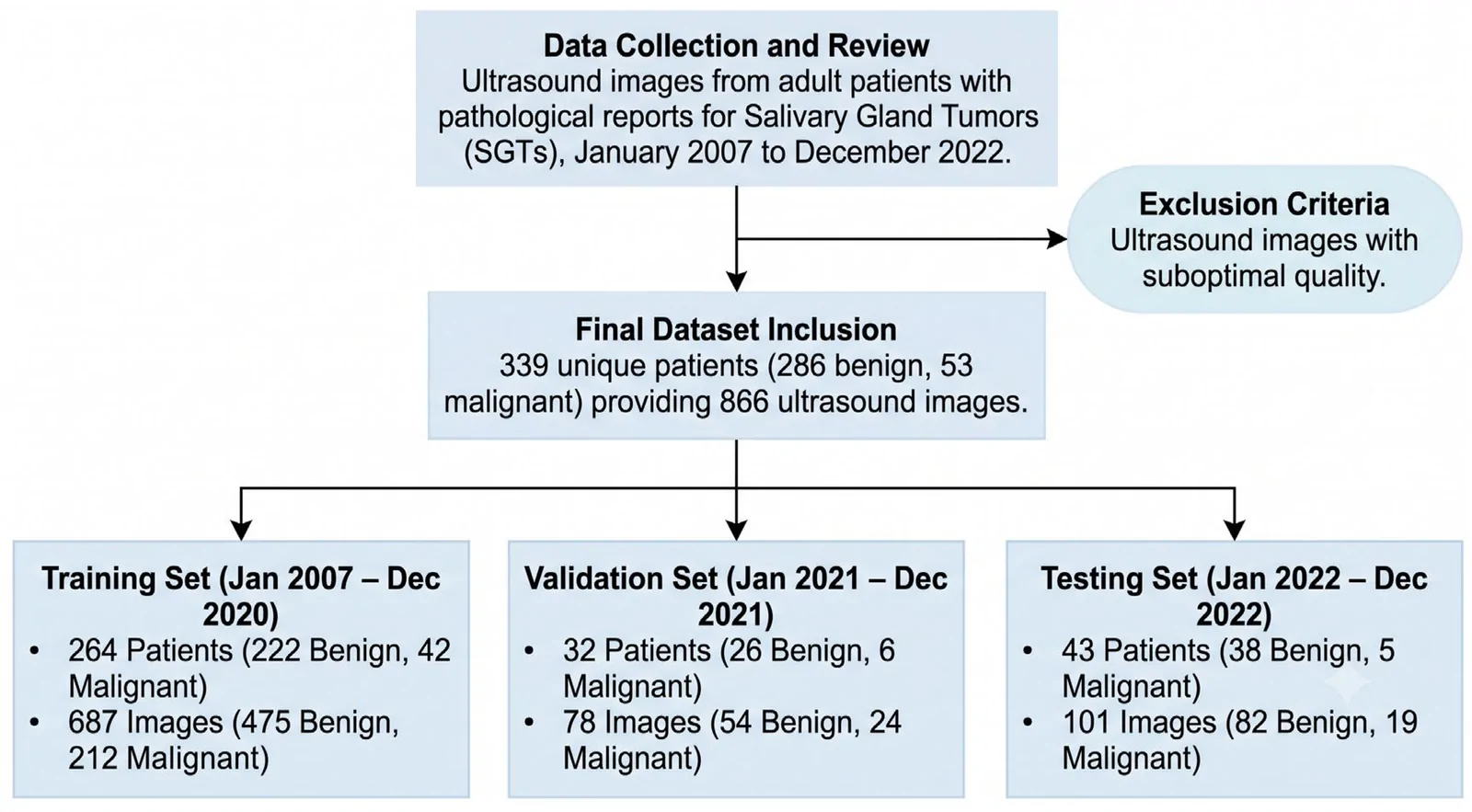
Flowchart to illustrate the study’s inclusion and exclusion criteria.

**Figure 2:**
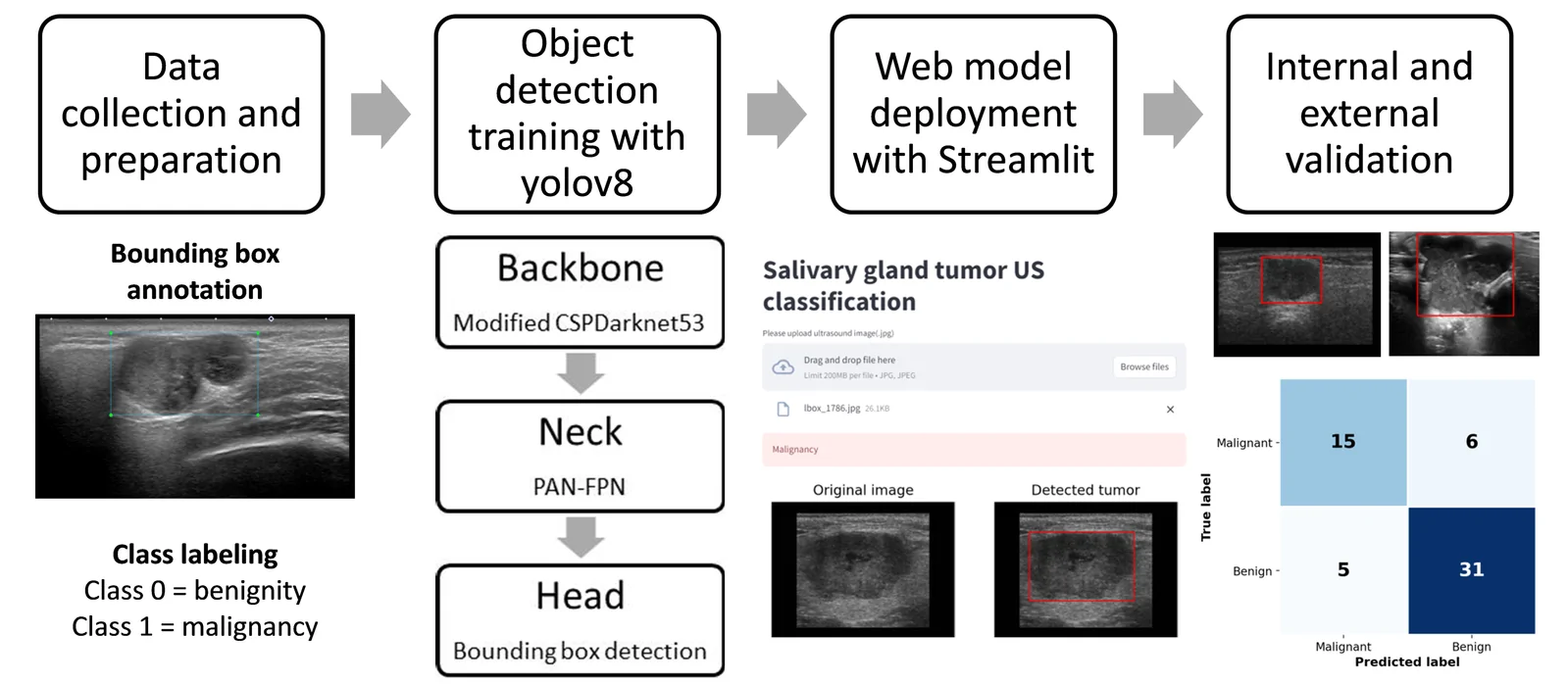
The study protocol. Abbreviation: PAN-FPN, path aggregation network with feature pyramid network.

**Figure 3:**
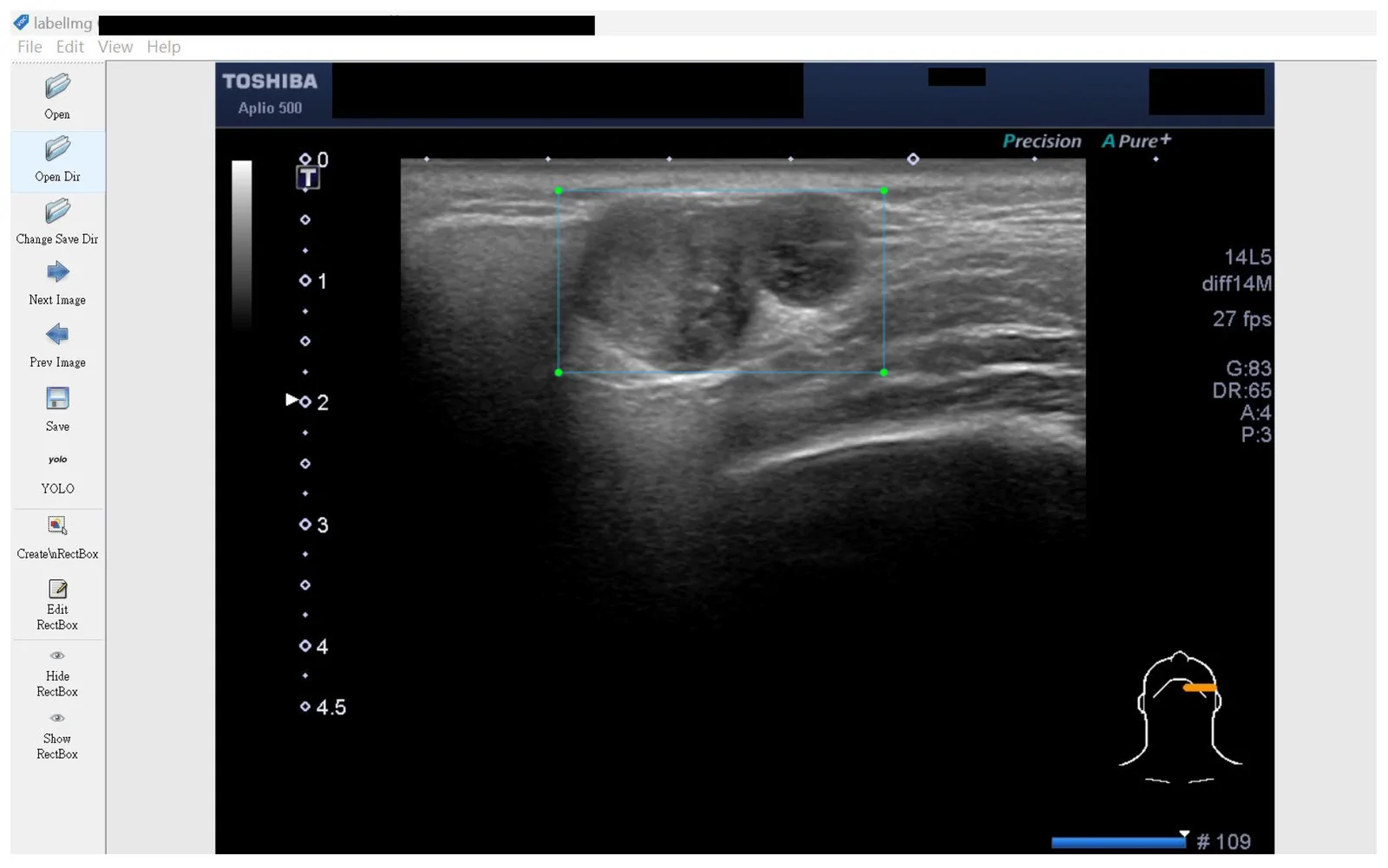
Bounding-box annotation using labelImg. Note: A rectangle bounding box was created using labelImg by experienced otolaryngologists.

The flowchart of inclusion and exclusion criteria is presented in Figure 1, and the study protocol is illustrated in Figure 2. A total of 866 ultrasound images were used for model development (training, validation, and testing), including 611 benign and 255 malignant cases. The dataset was temporally split into training (*n* = 687; 2007–2020), validation (*n* = 78; 2021), and testing (*n* = 101; 2022) sets. Using LabelImg, rectangular bounding boxes were manually drawn to tightly encompass the visible tumor margins in all 866 ultrasound images (Figure 3). All bounding boxes were subsequently cross-verified and approved by a third otolaryngologist. An additional 113 images were collected for independent evaluation, comprising internal (*n* = 56; 2023) and external (*n* = 57; online sources) validation cohorts.

**Figure 4:**
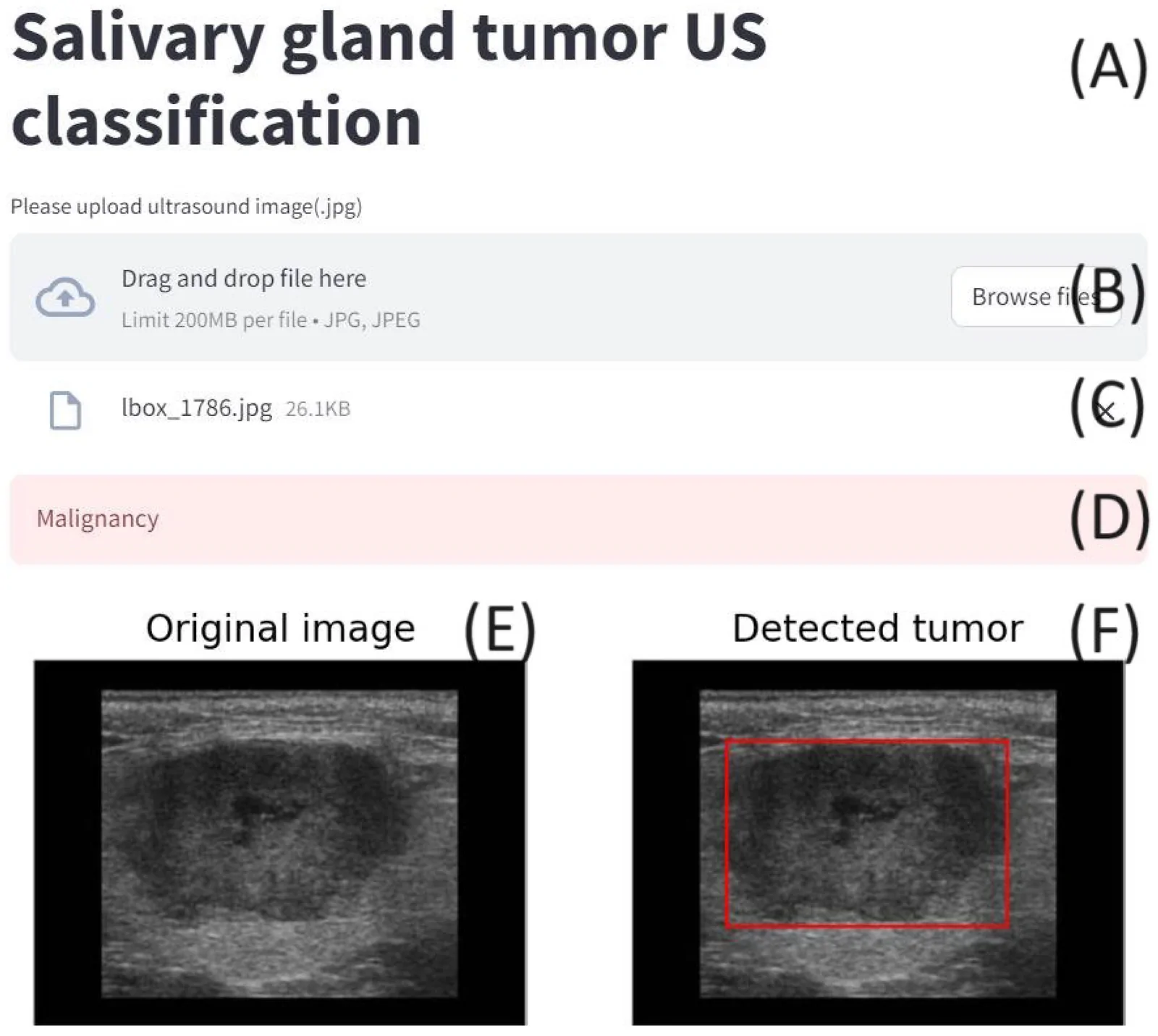
Web application interface. Note: The interface is structured vertically and includes the following elements: the title (**A**), an upload function (**B**), the filename of the uploaded image (**C**), the result of image classification (**D**), the uploaded image itself (**E**), and the detected tumor region highlighted (**F**). Abbreviation: US, ultrasound.

**Figure 5:**
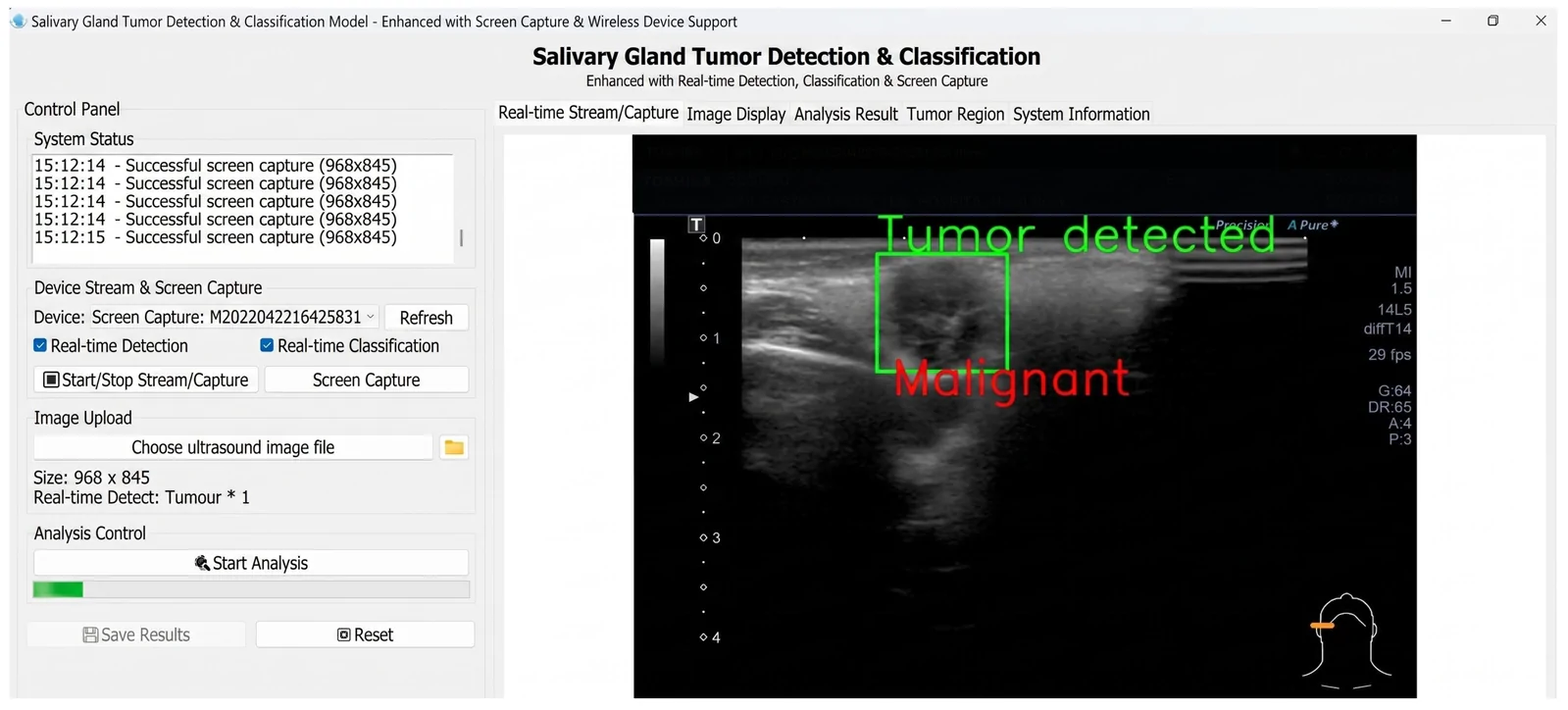
Desktop system interface. Note: The interface is organized into two panels. The left panel serves as the upload and control area. Users can integrate external streaming video from ultrasound machines, perform screen capture from external viewers, or upload videos and images for analysis. In streaming or video mode, users may choose whether to enable real-time detection and classification. If the edge device cannot support real-time processing, users can instead capture or upload a representative image for analysis. The right panel functions as the display area, where users can view the video or image along with the corresponding analysis results.

After 300 epochs of training, the YOLOv8 model achieved a bounding-box precision of 0.94, a recall of 0.95, and a mean average precision at IoU 0.5 (mAP50) of 0.96 on the testing set. The corresponding mAP50-95 was 0.69. A subsequent two-stage detection-and-classification pipeline was developed and deployed as both a web application (Figure 4) and a desktop system (Figure 5). This desktop system demonstrated an accuracy of 84%, a sensitivity of 74%, a specificity of 87%, a PPV of 56%, and an NPV of 93% on the testing set (Table 1). To evaluate deployment feasibility, we benchmarked the full desktop application on a representative edge device (Intel Core Ultra 5 125H CPU, 32 GB RAM, CPU-only inference). Excluding the first image (which includes a one-time model-loading overhead of 820 ms), mean latency was 132.9 ms/image for YOLOv8 detection (7.52 FPS), 82.1 ms/image for ResNet50V2 classification (12.19 FPS), and 223.5 ms/image end-to-end (4.47 FPS), confirming practical offline use on standard consumer hardware without a dedicated GPU.

**Figure 6:**
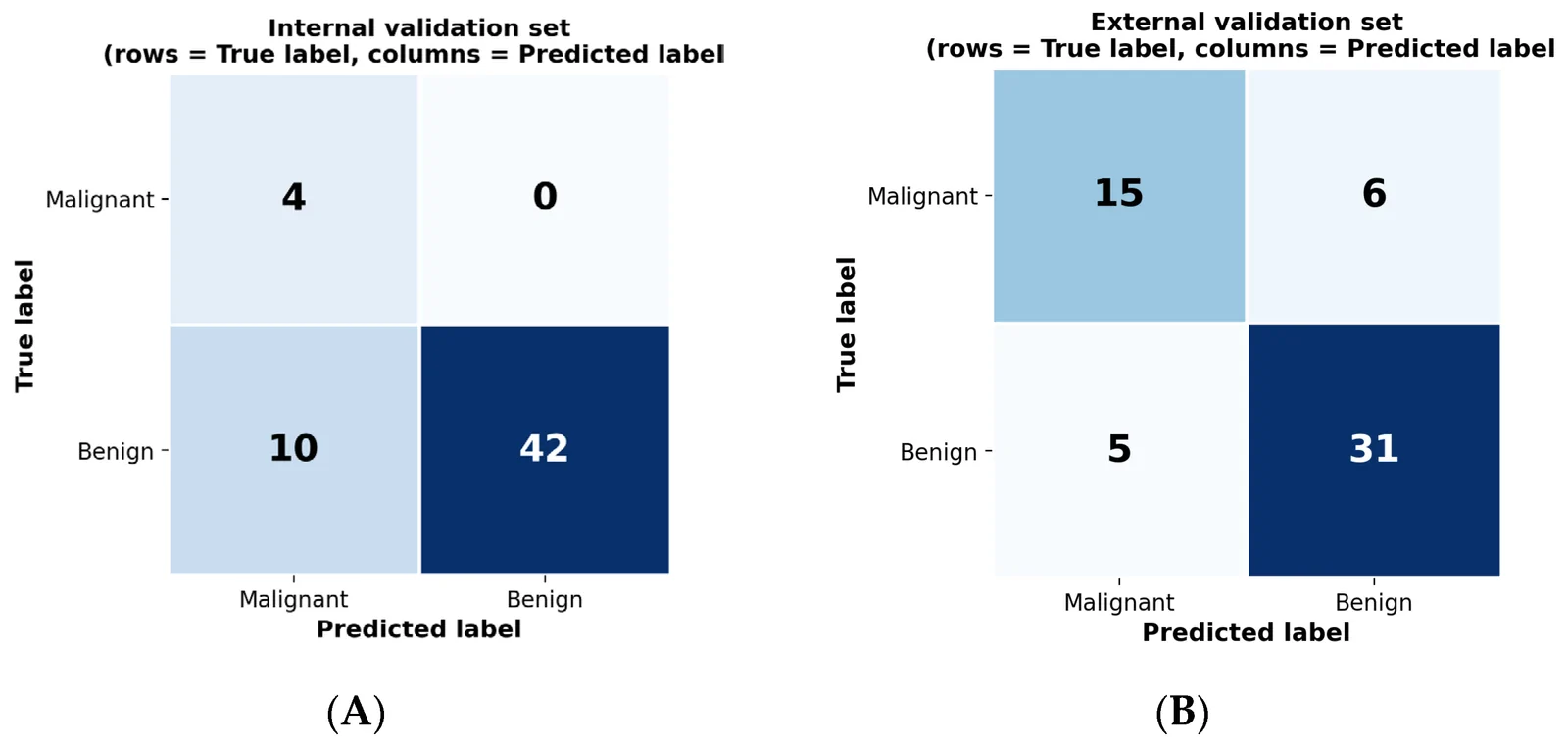
The confusion matrix for the internal validation set (**A**) and the external validation set (**B**), utilizing the desktop application system.

To evaluate clinical generalizability, both internal and external validations were performed. On the internal validation set, the model achieved an accuracy of 82%, sensitivity of 100%, specificity of 81%, PPV of 29%, and NPV of 100%. On the external validation set, it reported an accuracy of 81%, sensitivity of 71%, specificity of 86%, PPV of 75%, and NPV of 84%. The corresponding classification outcomes for these cohorts are illustrated in the confusion matrices (Figure 6 and Table 1). To further address statistical rigor, we computed ROC with AUC, precision–recall (PR) curves with AUC, balanced accuracy, and calibration analysis with Brier score using case-level probability outputs for all three cohorts (Table 1). Balanced accuracy was 80% for the testing set, 90% for internal validation, and 79% for external validation. ROC-AUC was 0.858 (testing), 0.889 (internal validation), and 0.791 (external validation). PR-AUC was 0.681 (testing), 0.379 (internal validation, reflecting the very small malignant sample, *n* = 4), and 0.748 (external validation). Unlike ROC-AUC, PR-AUC does not have a fixed 0.5 random-classifier baseline; instead, its baseline equals the malignant-class prevalence in each cohort. Brier scores were 0.143, 0.177, and 0.176 across three cohorts.

## 4. Discussion

This study represents one of the first successful attempts to engineer, optimize, and deploy an integrated, two-stage deep learning application for the automated detection and classification of SGTs using ultrasound images. By pairing a high-speed object detection model with an optimized CNN classifier, we eliminated the need for manual image preprocessing or human-directed ROI selection. This creates a direct raw-image-to-diagnosis pipeline suitable for clinical environments. The user-friendly web application and desktop system simplify the process for users by uploading a single ultrasound image of the SGT or video streaming. The primary contribution of this study is not the introduction of a novel network architecture but the development and validation of a deployable end-to-end workflow that integrates automated localization, classification, model compression, and offline edge deployment for SGT ultrasound analysis. Our desktop system demonstrated balanced diagnostic metrics on the testing set, achieving an accuracy of 84%, sensitivity of 74%, and specificity of 87%. When compared to the subjective ultrasound score [4], which exhibited a sensitivity of 58%, specificity of 89%, and accuracy of 85%, our desktop system showed an improvement in diagnostic sensitivity. Low sensitivity has historically been a key limitation of manual ultrasound interpretation and invasive cytology. Elevating this metric helps ensure fewer malignant lesions are missed during early screening. Beyond an automated diagnostic tool, the high-dimensional morphological and textural features extracted by our two-stage deep learning system can be conceptualized as a non-invasive digital biomarker. While traditional evaluation of SGT relies on invasive cytology or subjective ultrasound scoring, our optimized two-stage architecture acts as an objective digital assay that uncovers micro-structural tumor phenotypes imperceptible to the human eye [4]. Furthermore, the system maintained stable accuracies of 82% and 81% across both our temporal internal validation set and an independent international online dataset. This stability demonstrates that the application can successfully handle variations in image acquisition, patient demographics, and hardware configurations. The current system is a technically validated and deployable prototype, which can be used for tumor detection and classification in static images and videos (demonstration in Supplementary Video S1). Nonetheless, the wide confidence intervals observed, particularly in the internal validation cohort, reflect sample size constraints and warrant cautious interpretation. Consequently, the current system may serve as an objective adjunct to conventional ultrasound interpretation. Positive malignant predictions should serve to heighten clinical suspicion and guide further diagnostic workup, particularly for less experienced practitioners.

Beyond these threshold-based metrics, four complementary statistics reported in Table 1 (balanced accuracy, ROC-AUC, PR-AUC, and Brier score) together provide a more complete picture of the classifier’s behavior than any single metric alone. Balanced accuracy (80% testing, 90% internal, and 79% external) and ROC-AUC (0.858, 0.889, and 0.791) remained high and stable across cohorts, confirming robust discriminative ranking despite prevalence shifts. Conversely, PR-AUC varied considerably (0.681, 0.379, and 0.748) because its baseline is prevalence-dependent rather than fixed. The lower PR-AUC observed in the internal validation cohort likely reflects the extremely small number of malignant cases (*n* = 4), rather than a marked deterioration in discriminative ability. Brier scores (0.143–0.177) showed a similar pattern: while absolute values appeared comparable, internal validation calibration underperformed its baseline, whereas testing and external sets outperformed their baseline. Taken together, these four metrics indicate that the model’s ability to rank malignant cases above benign ones, reflected in ROC-AUC and balanced accuracy, is relatively robust to the prevalence differences across our three evaluation cohorts, whereas its precision and probability calibration, reflected in PR-AUC and Brier score, are considerably more sensitive to the very small malignant sample size in the internal validation cohort. This distinction has practical implications that the model’s relative case ranking may be reasonably trusted across settings, whereas its absolute predicted probability should be treated cautiously as a confidence score, particularly in low-prevalence settings.

To translate these models into practical clinical tools, different software development frameworks were evaluated for both web-based and local desktop deployment. For web deployment, two Python software development kits (SDKs), Gradio [13] and Streamlit [14], were evaluated for building interactive web components directly through Python code. Both frameworks are easy to develop and run on a local host; however, their online deployment mechanisms differ substantially. Gradio stores the deployed Python code on its own infrastructure, where projects are inherently public, posing potential challenges for medical image analysis due to privacy concerns. In contrast, Streamlit allows hosting code on GitHub, including private repositories, and deploys the web application under the Streamlit domain. Although the use of an external domain may still raise confidentiality concerns, clinicians and researchers can mitigate this by anonymizing ultrasound images prior to upload. For local system deployment, we utilized the Python Tkinter library. Tkinter, as a standard built-in Python graphical user interface (GUI) framework, was selected for its cross-platform compatibility and minimal external dependencies. It provides the intuitive interface required to integrate AI-generated outputs seamlessly into the clinical workflow. The OpenCV library functions as the intermediary layer, managing multiple image input sources and performing rapid preprocessing steps prior to model inference. Both deployment approaches demonstrated practical utility in clinical workflows. The web-based application provides a convenient solution for remote consultation; however, cloud-based deployment inherently introduces potential data transmission and privacy risks. In contrast, the local deployment system integrates directly with existing edge devices, ensuring that all patient-identifiable data remains securely within the hospital’s internal network.

In the field of object detection, models are generally categorized into one-stage and two-stage architectures. Although the two-stage object detection models are known for their superior accuracy, their slower processing speed limits their use in real-time object detection [15]. In our study, we employed the one-stage detection model, YOLOv8, for our detection training of SGT. When trained strictly for detection, it yielded impressive results in bounding-box prediction for SGTs, achieving a precision of 0.94, recall of 0.95, mAP50 of 0.96, and mAP50-95 of 0.69. YOLOv8 has been widely applied in medical image analysis, including brain tumor, breast cancer, and lung disease detection [16], and has also been extended to cytology and pathology imaging, where it achieves consistently high precision and recall [17]. Its capability for multi-class prediction further supports its use in detection-related tasks. We also evaluated different YOLO versions for detection, including YOLO11, which yielded inferior detection results and was therefore not pursued further. Although YOLO was created for detection and classification, in our preliminary experiments, using YOLOv8 for simultaneous detection and two-class (benign vs. malignant) classification resulted in suboptimal performance, achieving an overall mAP50 of 0.82 and mAP50-95 of 0.55. As an object detection framework, YOLOv8 simultaneously optimizes localization and classification, which may limit its ability to capture fine-grained, tumor-specific morphological features. A recent systematic review cataloged YOLO architectures (v1–v12) across diverse healthcare applications, underscoring the rapid evolution and clinical utility of this model family [18]. However, the authors also reported that relying solely on standalone YOLO models may lead to elevated false-positive and false-negative rates in automated diagnostic classification. To address this limitation, YOLOv8 was used solely as an automated cropping tool, while the subsequent classification task was delegated to a modified ResNet50V2 model. Although this two-model pipeline increases overall model complexity, the computational burden was mitigated using TFLite quantization. By converting model weights to 8-bit integers, file size and inference latency were significantly reduced, enabling efficient execution on standard non-GPU edge devices without compromising diagnostic performance. We note that “two-stage pipeline” here refers to our sequential localization-then-classification system (YOLOv8 followed by ResNet50V2), instead of “two-stage” as in the classic object-detection taxonomy (e.g., region-proposal-based detectors). We additionally compared classification accuracy using manually delineated, ground-truth tumor crops versus YOLOv8-predicted crops as ResNet50V2 input; manual cropping yielded modestly higher accuracy but cannot be automated for clinical deployment, so we consider this small trade-off justified by the gain in workflow automation. A paired ablation comparing pipelines with and without histogram equalization (HE) further showed that removing HE degraded performance across every metric in the external validation cohort (accuracy 80.7% → 64.9%; sensitivity 71.4% → 61.9%; specificity 86.1% → 66.7%) and reduced sensitivity in the testing set (73.7% → 57.9%), supporting the retention in HE as a preprocessing step for generalization to previously unseen image sources.

Despite these encouraging results, this study has several limitations. First, the retrospective design introduces potential selection bias, which may constrain the broader applicability of the findings. Second, the dataset utilized was relatively small in size, consisting of 866 ultrasound images of SGTs used for training, validation, and testing phases. Despite the implementation of distinct internal and external validation cohorts to assess the model’s diagnostic accuracy, the results derived from this specific dataset might not be generalizable to other demographic groups. Third, the low PPV (29%) in the internal validation set was driven by severe class imbalance, with only 4 malignant cases among 56 samples. In low-prevalence settings, even a highly sensitive model (100% sensitivity in this study) yields a low PPV, as the large number of benign cases naturally generates more false positives relative to true positives. Consequently, while the system’s high NPV renders it suitable as a screening tool, its false-positive rate warrants cautious interpretation, as a high false-positive rate could lead to unnecessary surgical referrals in clinical practice. Therefore, positive predictions from this system should serve to heighten clinical suspicion and prompt further diagnostic confirmation, particularly for less experienced practitioners. However, this finding should not be interpreted as evidence of poor model discrimination because PPV is strongly prevalence-dependent. Fourth, although we utilized histogram equalization to normalize our inputs, the system remains sensitive to variations in gray-level intensity. Significant differences in underlying ultrasound hardware settings or proprietary processing algorithms could still alter the grayscale distribution and affect classification outcomes. Fifth, formal inter-observer agreement for the bounding-box annotations was not quantified and should be incorporated into future annotation protocols. Sixth, although our benchmarking confirmed practical inference latency on standard CPU hardware, formal clinician usability testing and real-time streaming-mode throughput were not evaluated. We accordingly describe the current system as a technically validated, deployable prototype rather than a clinically validated tool. A multicenter validation study, which will also allow evaluation of domain adaptation across acquisition sites, is currently underway.

## 5. Conclusions

This study introduced an objective and automated approach to address the diagnostic challenges arising from subjectivity in ultrasound evaluation of SGTs. The application underwent rigorous evaluation and demonstrated strong diagnostic performance in both internal and external validation cohorts. By deploying this framework as both an accessible web interface and a secure offline desktop application, we show that the two-stage deep learning system can be effectively integrated into diverse clinical workflows. Our findings indicate that this two-stage deep learning system may serve as an objective adjunct to conventional ultrasound interpretation, potentially assisting clinicians during preoperative evaluation.

## Figures and Tables

**Table 1:** Diagnostic performance with 95% CI across the testing, internal validation, and external validation cohorts.

Set	ACC, % [95% CI]	SEN, % [95% CI]	SPE, % [95% CI]	PPV, % [95% CI]	NPV, % [95% CI]	Balanced Accuracy	Malignant Prevalence	ROC- AUC	PR- AUC	Brier Score
Testing (*n* = 101)	84% [76–90%]	74% [51–88%]	87% [78–92%]	56% [37–73%]	93% [86–97%]	80%	19% (19/101)	0.858	0.681	0.143
Internal validation (*n* = 56)	82% [70–90%]	100% [51–100%]	81% [68–89%]	29% [12–55%]	100% [92–100%]	90%	7% (4/56)	0.889	0.379	0.177
External validation (*n* = 57)	81% [69–89%]	71% [50–86%]	86% [71–94%]	75% [53–89%]	84% [69–92%]	79%	37% (21/57)	0.791	0.748	0.176

Abbreviations: ACC, accuracy; SEN, sensitivity; SPE, specificity; PPV, positive predictive value; NPV, negative predictive value; CI, confidence interval; ROC-AUC, receiver operating characteristic–area under the curve; PR-AUC, precision–recall area under the curve.

## Data Availability

The datasets analyzed during the current study are available from the corresponding author upon reasonable request.

## Supplementary Materials

The following supporting information can be downloaded at: https://www.mdpi.com/article/10.3390/biomedicines14081786/s1, Video S1: Demonstration of Automated Tumor Detection and Classification on Streaming Ultrasound Video.

## Abbreviations

The following abbreviations are used in this manuscript:

AUC	Area Under the Curve
CIoU	Complete Intersection over Union
CNB	Core Needle Biopsy
CNN	Convolutional Neural Network
DFL	Distribution Focal Loss
DL	Deep Learning
FNAC	Fine-Needle Aspiration Cytology
FPS	Frames Per Second
GPU	Graphics Processing Unit
GUI	Graphical User Interface
HE	Histogram Equalization
IoU	Intersection over Union
mAP50	Mean Average Precision at IoU 0.5
mAP50-95	Mean Average Precision at IoU Thresholds from 0.50 to 0.95
NPV	Negative Predictive Value
PACS	Picture Archiving and Communication System
PPV	Positive Predictive Value
PR	Precision-Recall
ROC	Receiver Operating Characteristic Curve
ResNet	Residual Network
ROI	Region of Interest
SDK	Software Development Kit
SGT	Salivary Gland Tumor
TFLite	TensorFlow Lite
YOLO	You Only Look Once
